# Application of the miRNAs as biomarkers and therapeutic strategies in periodontal inflammation

**DOI:** 10.3389/fphar.2026.1719006

**Published:** 2026-03-02

**Authors:** Sarmistha Saha, Nadezhda Sachivkina, Olga Pilshchikova, Alexandr Muraev, Sergey Ivanov, Sergey G. Ivashkevich, Regina Gurina

**Affiliations:** 1 Department of Biotechnology, Institute of Applied Sciences and Humanities, GLA University, Mathura, Uttar Pradesh, India; 2 Department of Microbiology V. S. Kiktenko, Institute of Medicine, Peoples’ Friendship University of Russia (RUDN University), Moscow, Russia; 3 Department of Therapeutic Dentistry, Institute of Medicine, Peoples’ Friendship University of Russia (RUDN University), Moscow, Russia; 4 Department of Oral and Maxillofacial Surgery, Institute of Medicine, Peoples’ Friendship University of Russia Named After Patrice Lumumba (RUDN University), Moscow, Russia; 5 Department of Maxillofacial Surgery, I.M. Sechenov First Moscow State Medical University, Moscow, Russia; 6 Agrarian and Technological Institute, Peoples’ Friendship University of Russia Named After Patrice Lumumba (RUDN University), Moscow, Russia

**Keywords:** inflammatory mediators, mir-142-3p, MiR-203, miRNA, periodontitis

## Abstract

Inflammatory and host immunological responses to bacterial tooth infections contribute to the development of periodontal disease. MiRNAs (miRNAs) play a critical role in regulating these immunological and inflammatory responses. miRNAs influence both innate and adaptive immunity in periodontal disease, affecting the functions of T and B cells, neutrophils, macrophages, and dendritic cells. This review examines the regulatory roles of miRNAs in periodontal tissues and evaluates their potential as therapeutic agents and biomarkers. Upregulated miRNAs identified include miR-146a, miR-29, miR-15, miR-148, and miR-223, while miR-31, miR-92, and miR-451 are downregulated. The review emphasizes the altered expression of miRNAs in periodontitis models. Selective targeting of miRNA pathways, enabled by gain or loss-of-function approaches, represents a promising strategy for therapeutic intervention. Additionally, the stability of miRNAs in gingival crevicular fluid and their association with specific features of periodontal disease underscore their potential as indicators of disease progression or tissue recovery.

## 1 Introduction

Periodontal disease is an inflammatory condition that affects the tissues surrounding the teeth (Cope et al., 2007). It arises from the immune system’s response to bacteria that infect these tissues. Clinically speaking, periodontal disease evolves from mild inflammation to severe tissue loss if treatment is not received. It varies between periods of disease activity and quiescence. The periodontium consists mainly of the gingiva, cementum (the outer layer of the tooth root), alveolar bone, which supports the teeth, and periodontal ligaments that connect the teeth to the alveolar bone (Nanci and Bosshardt, 2000). Plaque buildup stimulates the immune system, triggers an inflammatory response, and makes the gums more sensitive, which can occasionally result in bleeding. These plaques can lead to chronic inflammation and ongoing immune system stimulation if they are not eliminated (Nanci and Bosshardt, 2000). They also eventually harden and spread beneath the gum line. After these occurrences, the gum tissue progressively pulls away from the teeth, creating holes known as periodontal pockets, which allow bacteria to grow. This further strengthens the inflammatory process, and if it continues and inflammatory cytokines are continuously produced, osteoclastogenesis, tooth loosening, and ultimately tooth detachment may result (Harvey, 2017). *Porphyromonas gingivalis* (*Porphyromonas gingivalis*), *A. actinomycetemcomitans* (*Aggregatibacter actinomycetemcomitans*), and *Tannerella forsythia* (*T. forsythia*) are the most commonly identified bacteria linked to periodontitis (Van Dyke and Sheilesh, 2005). These bacteria possess certain virulence factors, such as fimbriae, adhesins, lipopolysaccharides, hemagglutinins, proteinases, and toxic metabolites, which facilitate their survival and proliferation (Shahoum et al., 2023). Recognizing and effectively addressing the severity of periodontitis is crucial for enhancing public health and overall wellbeing, and proactive measures can significantly contribute to preventing its impact.

The current classification system identifies stages III and IV of periodontitis as particularly challenging yet important to address. These advanced stages are marked by angular bone defects, furcation involvement, tooth mobility, significant tooth loss, and a decline in functionality. At this point, a variety of intrinsic and environmental risk factors can impact the host’s capacity to effectively combat bacterial infections and manage tissue damage. While only 10%–12% of individuals are affected by severe periodontitis, it often affects multiple teeth in each person, highlighting the importance of early intervention and management (Billings et al., 2018; Needleman et al., 2018). The progression of periodontitis is primarily linked to an abnormal immune response to the subgingival plaque biofilm. Understanding this intricate relationship, which sees bacterial infections in dental plaque trigger host cells to release a cascade of pro-inflammatory cytokines, is crucial. This immune response also leads to an oxidative burst, resulting in an excess of reactive oxygen species and proteolytic enzymes (Hajishengallis et al., 2000). By focusing on prevention and tailored treatment strategies, we can enhance our ability to manage and mitigate the effects of severe periodontitis, ultimately improving oral health outcomes.

Periodontal progenitor cells (PDLSCs) serve as vital tissue-specific stem cells that contribute to the production of new periodontal ligaments (PDL) and play a key role in maintaining the balance within periodontal tissue (Dangaria et al., 2011). These essential cells derive from neural crest-derived intermediate progenitors in the dental follicle, which differentiate into PDL fibroblasts, alveolar bone osteoblasts, and cementoblasts (Luan et al., 2009; Da et al., 2011). In addition to supporting the nonmineralized PDL, PDLSCs are crucial in preserving the integrity of the mineralized alveolar socket, which anchors teeth firmly in the jaws (Jung et al., 2011). Research utilizing the early mineralization marker RunX2 has demonstrated that mammalian periodontal progenitors originate from a shared lineage associated with mineralized tissue. This finding underscores the importance of precisely controlled spatial mineralization for the effective differentiation of periodontal tissues (Luan et al., 2006). Such insights pave the way for further advancements in understanding periodontal health and developing targeted therapeutic strategies.

MiRNAs (miRNAs) are essential short non-coding RNA molecules, typically measuring between 19 and 24 nucleotides. They effectively regulate gene expression by either inhibiting translation or destabilizing mRNA through specific binding sites in the 3′untranslated region (UTR) of genes (Luan et al., 2017). By playing a key role in post-transcriptional gene regulation, miRNAs significantly influence a wide array of biological processes in human cells, which are vital for DNA transcription and protein synthesis (Laberge et al., 2023). miRNAs serve as important epigenetic regulators of gene expression, significantly impacting several cellular functions such as apoptosis, differentiation, and cell proliferation (Larsson et al., 2022). Their critical role in the development of diseases and the inflammatory response highlights the potential for targeting miRNAs in therapeutic applications and enhances our understanding of complex biological processes.

**FIGURE 1:**
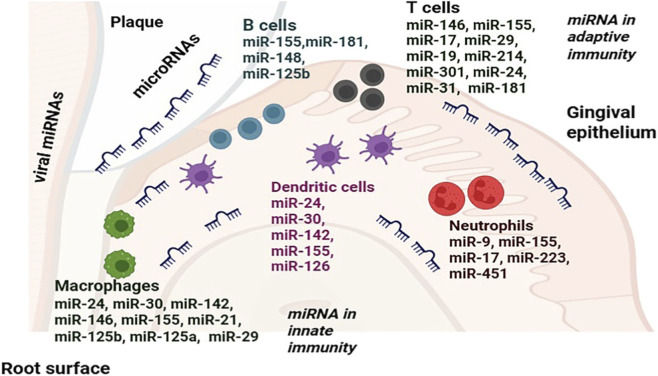
Schematic diagram illustrates that miRNAs regulate both innate and adaptive immune responses in the periodontium. The immune system within the periodontium is activated by bacterial plaque present on the enamel surface and within the gingival sulcus. miRNAs modulate the function of innate immune cells, including neutrophils, dendritic cells, and macrophages, as well as adaptive immune cells, such as T and B lymphocytes, by targeting specific genes.

The primary transcripts of miRNAs, known as pri-miRNAs, are produced from specific coding sequences in mammalian cells. The Drosha/DGCR8 complex efficiently processes most pri-miRNAs into short hairpin RNAs, or precursor miRNAs, which are then cleaved by Dicer into miRNA duplexes ranging from 21 to 25 nucleotides in length. This process is essential for the assembly of the RNA-induced silencing complex (RISC), which targets complementary mRNAs for gene silencing by incorporating the mature miRNA strand (Suzuki and Miyazono, 2011). The latest update from MIRBASE (version 21) highlights the identification of 1,881 precursor miRNAs and 2,588 mature miRNAs in humans, showcasing the rich diversity and potential of these molecules (Griffiths-Jones et al., 2008). Before reaching their target cells, miRNAs are often released into the extracellular space through an exosome pathway (Zhang et al., 2015). While intracellular miRNAs are crucial for regulating gene expression and protein translation, extracellular miRNAs serve an even broader role by facilitating cell communication (O’Brien SJ. et al., 2018). Emerging research highlights that miRNA are vital components of exosomes derived from mesenchymal stem cells, playing a pivotal role in controlling a wide array of cellular signaling pathways. These compelling findings underscore the potential of miRNAs as a formidable mechanism for bone regeneration in periodontal ligament stem cells (PDLSCs). In this study, we are highlighting the most important elements of miRNA function that are currently understood in relation to periodontal tissue homeostasis and disease progression. Gingival epithelial cells (GECs) are crucial for periodontal health, enhancing innate immunity and serving as a barrier against harmful microorganisms (Dale, 2000). They release pro-inflammatory mediators in response to microbial agents and are constantly exposed to bacterial products. Through the expression of miRNAs, human gingival epithelial cells (HGECs) play a key role in regulating periodontal inflammation (Figure 1).

Dysregulated miRNA expression in tissues is also observed in biofluids such as serum, saliva, and gingival crevicular fluid (Turchinovich and Cho, 2014). Consequently, miRNAs serve as sensitive and specific biomarkers for various diseases. Because cell types respond differently to the same pathogen, the role of miRNAs during host responses to bacterial infections depends on the cellular context (Maudet et al., 2014; Taheri et al., 2024). Regulating miRNA expression through gain or loss of function techniques allows for selective targeting of miRNA pathways, offering a promising strategy for therapeutic intervention in diverse diseases.

An overview of the miRNAs in gingival and periodontal cells and tissues, their role in periodontal infection and inflammation, and how miRNAs work together to coordinate the periodontal immune response, strong enough to repel bacterial and viral aggressors while being mild enough to prevent an increase in inflammation and tissue loss will be given in this review. Our review will examine all elements of the miRNA response to periodontal pathogens, including the control of oral microbial pathogens that originate and sustain periodontal disease and the genetic and host microenvironmental factors that determine the degree of the diseases. A summary of the primary sites and mechanisms *via* which miRNAs regulate periodontal immunity and tissue homeostasis during periodontal inflammation are given in Figure 1.

## 2 miRNAs in periodontal innate immunity

Recent advancements in biomedical science have propelled our understanding of inflammatory diseases, particularly periodontal disease. Numerous studies have revealed that miRNAs (miRNAs) play a critical role in regulating inflammation and shaping immune responses. Notably, specific miRNAs have been identified as key contributors to the development of periodontitis, highlighting their potential as targets for innovative therapeutic interventions (Table 1) (Luan et al., 2022; Yue et al., 2016). Because it contributes to and functionalizes cells in the first host response against infection, the first stage of defense against oral bacteria is known as the innate immune response. Neutrophils are the most significant phagocytic cells among these 3 cell types, and they are the main players in the host’s defense against an acute bacterial infection. Neutrophil miRNAs that are functionally significant include miR-155 and miR-223, which are elevated in periodontal disease, and miR-17 and miR-31, which are downregulated.

**TABLE 1:** Summary of miRNAs roles in the pathogenesis of periodontal inflammation.

Immune cells	miRNAs	Target Gene	miRNA expression in periodontal disease tissues	References
B cells	miR-155	*Activation-Induced Cytidine Deaminase (AID)*	Up	Luck et al. (2015)
	miR-148	*PTEN, Bach2, Bim*	Up	Takahashi et al. (2012), Akkouch et al. (2019)
	miR-125b	*Blimp1, IRF4*	Up	Pegtel and Gould (2019)
T cells	miR-146	*IRAK1*	Up	Atsawasuwan et al. (2018)
	miR-155	*SOCS1*	Up	Shen et al. (2020)
	miR-181	*SHP2, PTPN22, DUSPS5*	Up	Nik Mohamed Kamal NNS et al. (2020), Lv et al. (2020)
	miR-301	*PIAS3*	Up	Han et al. (2020)
	miR-24	*FoxP3*	Up	Krongbaramee et al. (2021), Lu et al. (2023)
	miR-17	*KZF4, CREB1, TGFβRII*	Up	Xia et al. (2025), Lin et al. (2022)
	miR-214	AKT STAT3, STAT2	Down	Kwon et al. (2023)
Dendritic cells	miR-155	*c-Fos*	Up	Wang et al. (2016)
	miR-142	*PKCα, TLR4, CD14*	Up	Xie et al. (2011), Lundmark et al. (2018)
	miR-126	*TSC-1*	Up	Kang et al. (2023)
	miR-30	*PKCα, TLR4, CD14*	Up	Lundmark et al. (2018)
	miR-24	*PKCα, TLR4, CD14*	Up	Lundmark et al. (2018)
Macrophages	miR-21	*STAT3*	Up	Wei et al. (2016)
	miR-24	*PKCα, TLR4, CD14*	Up	Lundmark et al. (2018)
	miR-30	*PKCα, TLR4, CD14*	Up	Lundmark et al. (2018)
	miR-29	*IL-6Rα*	Up	Cubillos-Ruiz et al. (2012), Zhang et al. (2013)
	miR-125b	*IRF4*	Up	Yang et al. (2015), Mishra et al. (2016)
	miR-146	*TRAF6,* SMAD4, *IRAK1, IRAK2*	Up	Xavier et al. (2015), Gaharwar et al. (2014)
	miR-155	*MAP3K10, IL13Rα1, BCL6, SMAD2*	Up	Munagala et al. (2016), Wang et al. (2025), Wang et al. (2020b), Diener et al. (2022), Sharma et al. (2022)
	miR-147	TLRs signaling	Up	Stoecklin-Wasmer et al. (2012)
	miR-200b and miR-200c	*MyD88, TLR4, NF-κB*	Up	Qin et al. (2013)
Neutrophil	miR-17	*ICAM-1*	Up	Li and Kowdley (2012)
	miR-34	*Dock8*	Up	Cheng et al. (2014)
	miR-155	*SHIP-1, FGD4*	Up	Cheng et al. (2014), Chen et al. (2008), Mitchell et al. (2008)
	miR-223	*CXCL2, CCL3, IL-6*	Up	Robbins and Morelli (2014)
	miR-451	CPNE3, Rab5α	Down	Fernández-Messina et al. (2015)

IL-8, a member of the CXC chemokine subfamily, plays a vital role in attracting neutrophils to areas of damage or infection. It appears that IL-8 is another direct target of miR-181a (Galicia et al., 2014). Consequently, miR-181a may slow the development of oral pulp inflammation, potentially *via* lowering IL8 expression; in fact, it may stop pulpitis from developing into periodontitis. It appears that miR-17 can lower the expression of IL-8 in neutrophils by targeting SHIP1 but miR155 induces overexpression of IL-8 by blocking its expression (Gantier, 2013). The expression of miR-451 in neutrophils appears to reduce neutrophil migration to the infection site and inhibits the expression of inflammatory factors such as cyclooxygenase-2, TNF-α, and IL-1β (Murata et al., 2014).

**FIGURE 2:**
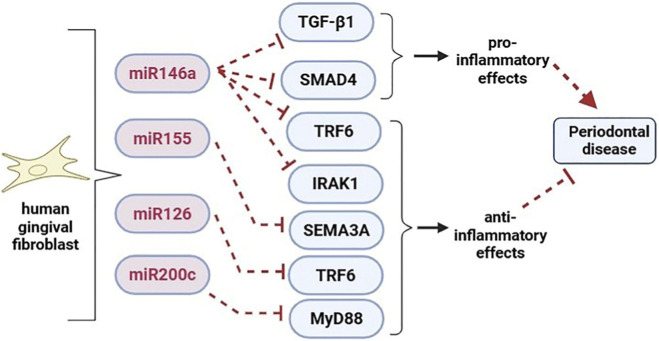
In periodontal fibroblast cells, miRNAs have both pro- and anti-inflammatory actions.

miR-203 emerges as a pivotal miRNA. This particular miRNA appears to enhance cytokine production, thus playing a significant role in the advancement of periodontitis. It achieves this by inhibiting the suppressor of cytokine signaling 3 (SOCS3), which is crucial for maintaining the integrity of the innate immune response (Moffatt and Lamont, 2011). Moreover, periodontitis also correlates with other miRNAs, including miR-155, miR-126, and miR-210 (Figure 2) (Stoecklin-Wasmer et al., 2012). There is a possibility that GECs express these miRNAs, which work to modulate inflammatory responses to microbial infections. Notably, miR-126 encourages the overproduction of important chemokines, such as C-C motif chemokine ligand 1 (CXCL1) and interleukin-8 (IL-8), thereby facilitating the attraction of immune cells, particularly neutrophils. On the other hand, miR-155 and miR-210 play a role in regulating the expression of IL-8 and CXCL1, providing a balanced approach to inflammation (Chen et al., 2016).

The expression and effects of miR-146a and miR-155 in fibroblasts have been the subject of several investigations. Human gingival fibroblasts (HGFs) activated by *P. gingivalis* LPS appear to express miR-146a at a considerably higher level than HGFs that are not stimulated. miR-146a suppresses the release of pro-inflammatory cytokines like IL-6 and TNF-α in HGFs, presumably by lowering IRAK1 expression (Bhaumik et al., 2009). However, miR-155 expression in HGFs decreases in pro-inflammatory circumstances. According to a study, miR-146a inhibits the production of TNF receptor related factor 6 (TRAF6), which in turn inhibits the secretion of IL-8, IL-6, and IL-1β in HPDL fibroblasts stimulated by *Porphyromonas gingivialis* (Tang et al., 2019). Another miRNA that targets TRAF6 is miR-126. According to a study on miR-126’s impact on fibroblasts, miR-126 suppresses the release of pro-inflammatory cytokines such as IL-6, TNF-α, and C-C Motif Chemokine Ligand 2 (CCL2) in HGFs by decreasing the expression of TRAF6 and so attenuating NF-κB activity (Wu et al., 2017). Furthermore, in hPDLCs, overexpression of miR-146a markedly reduced the production of the inflammatory cytokines TRAF6 and IRAK1 (Fumimoto et al., 2024).

In response to LPS, macrophages in inflammatory periodontal tissues produce inflammatory mediators that trigger several host defensive mechanisms (Luan et al., 2018). MiRNA networks, which are made up of individual miRNAs like miR-24, miR-30b, and miR-142–3p that have antagonistic effects against periodontal inflammation and inflammation-related tissue destruction (Luan et al., 2018), finely regulate macrophage differentiation, activation, and response to LPS (Roy, 2016; Zhou et al., 2016). The inflammatory miRNAs miR-146a and miR-155, which are involved in the activation of macrophages in inflammatory gingival tissues, and additional periodontal miRNAs involved in the macrophage response are let-7f and miR-29b (Stoecklin-Wasmer et al., 2012; Xie et al., 2011; Jiang et al., 2015; O’Connell et al., 2007; Taganov et al., 2006).

In another study, miR-214 was further examined through overexpression and silencing (Cao et al., 2017). Because miR-214’s overexpression reduced osteogenesis while its knockdown had the opposite effect, the results suggested that miR-214 may be involved in the mechanisms underpinning PDLSCs’ osteoblastic differentiation. Additionally, miR-214 was shown to directly target the β-catenin gene CTNNB1, and it was shown that miR-214 promotes osteoblastic development *via* modifying Wnt/β-catenin signaling. The findings of this study also indicated that miR-214 may participate in the mechanisms underpinning PDLSC development *via* regulating the Wnt/β-catenin signaling pathway by targeting CTNNB1. According to results from a different study, miRNAs regulate osteogenic differentiation from hPDLSCs in a unique way. Specifically, miR-214 reduces osteogenic differentiation of hPDLSCs by targeting ATF4 (Yao et al., 2017).

Through the activation of the signaling cascade triggered by TLR2/4 and NF-κB, *P. gingivalis* causes macrophages to overexpress miR-132, which excites the production of TNFα. In response to *P. gingivalis* LPS-induced NF-κB activation, macrophages can additionally express miR-21 (Park et al., 2016). By specifically targeting PDCD4, miR-21 can reduce NF-κB activity and hence suppress macrophage production of proinflammatory cytokines. By inhibiting STAT3, this miRNA also reduces macrophage polarization to the M2 phenotype (Xi et al., 2018). It appears that M1 macrophage polarization is linked to the overexpression of miR-147 in periodontal tissue, and that this miRNA can increase the production of M1 macrophage markers such as TNF-α, IL-12, and Nos2 (Xi et al., 2018). An additional investigation was conducted to examine the role of TLR2/4 signalling in the miRNA profile following polybacterial infection in global knockout strains of TLR2−/− and TLR4−/− mice and C57BL6/J wild-type mice. TLR2 knockout and wild-type mice have increased levels of miR-15a-5p. Let-7c-5p was downregulated in wild-type mice and increased in TLR4−/−mice (Jeepipalli et al., 2025a). According to this study, wild-type infected mice had increased levels of miR-146a-5p, miR-15a-5p, and miR-132-3p. TLR2−/− infected mice had increased levels of miR-146a-5p and miR-15a-5p. The TLR4−/− infected animals showed upregulation of seven miRNAs: miR-30c-5p, miR-22-5p, miR-323-3p, miR-361-5p, miR-375-3p, miR-720, and let-7c-5p (Jeepipalli et al., 2025a).

Dendritic cells demonstrate the expression of miR-142-3p, which plays a crucial role in modulating their production of inflammatory mediators. By controlling the gene expression of transcription factor networks, miRNAs influence both primary and secondary immune responses in dendritic cells (Luan et al., 2018). Numerous dendritic cell-related miRNAs, such as miR-24, miR-30, miR-126, miR-142, miR-146, miR-155, and let-7i, were found to be increased in a mouse model of periodontitis in earlier research, and published human investigations supported the involvement of these miRNAs in periodontal disease (Luan et al., 2018). When exposed to LPS, these cells enhance the expression of miR-142-3p, which effectively targets the IL-6 transcript. This process contributes to a significant reduction in the intensity of inflammation, highlighting the potential of miR-142-3p as a key regulator in inflammatory responses (Sun et al., 2011).

Research has revealed some intriguing roles of miR-155 in the regulation of the TLR4 signaling pathway. Notably, miR-155 inhibits the expression of certain inhibitory molecules, including signaling suppressor of cytokine-1 (SOCS-1) and SH2 domain-containing inositol 5′-phosphate 1 (SHIP1). This action promotes the activation of the TLR4 signaling pathway and NF-κB, leading to an increased expression of IL-8, which is associated with inflammation development and progression (Kanwal et al., 2013). Furthermore, additional studies indicate that miR-155 can also play a protective role by attenuating this signaling pathway. It does this by downregulating molecules that reinforce TLR signaling, such as TAK1-binding protein 2 (Table 2), Myeloid differentiation primary response 88 (MyD88), and IκB kinase (IKK). It appears that IFN-β-induced overexpression of miR-155 inhibits osteoclast development by targeting SOCS-1 and microphthalmia-associated transcription factor (MITF), two key regulators of osteoclastogenesis (Zhang et al., 2012). Another miRNA linked to periodontal disease is miR-142. It appears that HGECs’ expression of miR-142 rises sharply in response to the inflammation brought on by TNF-α (Li et al., 2017). It appears that decreased expression of IL-8 is linked to miR-17 expression (Oglesby et al., 2015).

**TABLE 2:** Summary of miRNAs expressions in gingival tissues of periodontitis animal models and human patients compared to corresponding controls.

miRNAs	Expressions
miRNA expression profile in gingival tissues of periodontitis animal models (Xi et al., 2018; Rolfes et al., 2025; Jeepipalli et al., 2025b; Aravindraja et al., 2024; Aravindraja et al., 2023a; Aravindraja et al., 2023b; Aravindraja et al., 2023c)	
miR-21a	Upregulated
miR-22-3p	Upregulated
miR-203	Upregulated
miR-26a	Upregulated
miR-29b	Upregulated
miR-30d	Upregulated
miR-103	Upregulated
miR-125a	Upregulated
miR-126-3p	Upregulated
miR-126-5p	Upregulated
miR-146a and b	Upregulated
miR-15a-5p	Upregulated
miR-181b	Upregulated
miR-221	Upregulated
miR-223	Upregulated
miR-361-5p	Upregulated
miR-99b-5p	Upregulated
miR-146a	Upregulated
miR-206	Upregulated
miR-210	Upregulated
miR-151a-3p	Upregulated
miR-152	Upregulated
miR-125b-5p	Upregulated
miR-133a	Upregulated
miR-34b-5p	Upregulated
miR-486	Upregulated
miR-378a-3p	Upregulated
miR-690	Upregulated
miR-804	Upregulated
miR-1902	Upregulated
miR-423-5p	Upregulated
let-7c-5p	Upregulated
miR-17	Downregulated
miR-24	Downregulated
miR-15a-5p	Downregulated
miR-27a-3p	Downregulated
miR-30	Downregulated
miR-34b-5p	Downregulated
miR-92a	Downregulated
miR-133	Downregulated
miR-1224	Downregulated
miR-451	Downregulated
miR-362-3p	Downregulated
miR-720	Downregulated
miR-375	Downregulated
miR-376	Downregulated
miR-376a	Downregulated
miR-350	Downregulated
miR-323-3p	Downregulated
miR-302b	Downregulated
miR-488	Downregulated
miR-1902	Downregulated
miR-1937	Downregulated
miR-1937a	Downregulated
miR-2135	Downregulated
miRNA expression profile in gingival tissues of periodontitis patients (Rapado-González et al., 2018; Lee et al., 2011; Stoecklin-Wasmer et al., 2012; Xie et al., 2011)	
miR-17	Upregulated
miR-19a,b	Upregulated
miR-26	Upregulated
miR-29a,b,c	Upregulated
miR-30b,c,d	Upregulated
miR-34a,c	Upregulated
miR-30b,c,d	Upregulated
miR-34a,c	Upregulated
miR-126	Upregulated
miR-142	Upregulated
miR-146a	Upregulated
miR-148	Upregulated
miR-155	Upregulated
miR-223	Upregulated
miR-301	Upregulated
miR-31	Downregulated
miR-92a	Downregulated
miR-214	Downregulated
miR-451	Downregulated

## 3 miRNAs in adaptive immune modulation

An acquired immune response to invasive infections is known as adaptive immunity. Adaptive immunity starts with a trigger event that causes the immune system to recognize a pathogen. This is followed by an increased immune response when the pathogen is encountered again (Wang R. et al., 2020). Adaptive immunity comes in two flavors: cell-mediated immune responses against intracellular pathogens, which are executed by T cells, and antibody-mediated immune responses against freely circulating pathogens, which are assisted by B cells (Wang R. et al., 2020).

T cells are fundamental components of the cellular (macrophage/lymphocyte) immune response and are necessary for the stimulation of polyclonal B cells as well as the synthesis of certain antibodies. T-cell receptors are found on the surface of T-cells. It has been shown that upregulation of the miR-214 and miR-17–92 cluster promotes T cell activation and proliferation, while miR-146 acts as a feedback regulator of NF-κB signaling and modulates the productive immune response, division, and growth of T cells (Yang et al., 2012). MiRNAs also have an indirect impact on T cell function. Thus far, it has been demonstrated that three miRNAs - miR-155, let-7 family members, and miR-126 that affect Th2 differentiation *via* controlling the synthesis of cytokines (Podshivalova and Salomon, 2013).

Periodontitis has been linked to a Th17/Treg imbalance (Iniesta et al., 2023). Th17 cells induce inflammation, while Treg cells decrease it (Jia et al., 2024). Th17 cells express RORγt, a transcription factor that produces the proinflammatory cytokine IL-17, which contributes to immune-mediated illnesses and tissue damage (Nagata et al., 2023). Treg cells express Foxp3 and secrete anti-inflammatory cytokines, including TGF-β and IL-10, which restore immunological balance and reduce tissue damage (Zhang et al., 2023; Gao et al., 2023). To summarize, the Th17/Treg imbalance is critical for the development of periodontitis; thus, maintaining this balance offers a novel strategy for periodontal treatment. Many miRNAs play a role in controlling the balance of Th17 and Treg cells. It has been suggested that a key element in the pathophysiology of periodontitis is the functional antagonistic relationship between regulatory T cells (Treg cells) and T helper 17 cells (Th17), which produce IL-17 (Diekwisch, 2016). In order to contribute to and intensify the inflammatory response during periodontal disease, Th17 cells release IL-17 and attract neutrophils and macrophages (Allam et al., 2011; Zhao et al., 2011; Laurence and O’Shea, 2007). High levels of miR-155 and miR-146 and low levels of miR-24, miR-31, and miR-125 expression are characteristics of Treg cells (Rouas et al., 2009; Sethi et al., 2013). On the other hand, the miR-17–92 cluster member miR-19b (Liu et al., 2014), together with miR-301 and miR-155, stimulate Th17 cell differentiation. Previous studies show that miRNAs play a role in Treg/Th17 functional antagonism during periodontal disease progression, with upregulation of miR-17, miR-19, miR-155, and miR-301 in gingival tissues of patients and animals compared to healthy controls (Xie et al., 2011; Lee et al., 2011; Stoecklin-Wasmer et al., 2012).

In Th1 and Treg cells, miR-146a is substantially expressed (Cobb et al., 2006). By focusing on the Fas-associated death domain, this miRNA can prevent cell death and shield T cells from activation-induced cell death (Curtale et al., 2010). One important factor in the reduction of the Th1 response is the expression of miR-146a in Treg cells. STAT1 is one of miR-146a’s targets. STAT1 is required for Th1 cell differentiation. It appears that miR-146a targets STAT1 to enhance Treg cells’ ability to regulate the Th1 response (Testa et al., 2017). By focusing on diverse parameters, miR-155 can encourage the development of Th1 cells, Th17 cells, and Treg cells (Chen et al., 2020). Additionally, when CD8^+^ T cells are activated, miR-155 expression is similarly elevated, but it rapidly declines (Jeker and Bluestone, 2013).

Additional miRNAs linked to Treg cells include miR-17, miR-126, and miR-142-3p. By targeting TGFβRII and cAMP-responsive element-binding protein1 (CREB1), miR-17 appears to have the potential to inhibit the development of iTreg cells. Treg cells exhibit heightened expression of miR-126, and their inhibitory action is reduced when it is absent (Jiang et al., 2011). Absence of this miRNA appears to increase p58B production, which in turn promotes the PI3K/Akt pathway. Subsequently, the activation of this pathway results in a modification of Treg cells’ inhibitory function by decreasing Foxp3 expression (Qin et al., 2013). Foxp3 appears to be able to lower miR-142-3p expression in Treg cells. After activation, this miRNA may inhibit Treg cell proliferation (Phiwpan and Zhou, 2018).

MiR-29 plays a pivotal role in regulating Th1 cell differentiation and IFN-γ production by targeting the transcription factors Eomesodermin (EOMES) and T-bet. By doing so, miR-29 effectively suppresses IFN-γ production in Th1 cells, and it directly influences the transcription of IFN-γ in T cells, which further inhibits Th1 cell development (Smith et al., 2012). This regulatory mechanism highlights the importance of miR-29 in maintaining balance within the immune response. In addition, human regulatory T (Treg) cells exhibit increased expression of miR-21, which serves as a positive regulator of FOXP3 expression, thereby supporting Treg cell function and stability (Rouas et al., 2009). Furthermore, miR-181a is significant in promoting T cell responses. It enhances T cell proliferation and differentiation by upregulating molecules involved in the T cell receptor (TCR) signaling pathway while simultaneously blocking negative regulators such as DUSP6 phosphatase (Li et al., 2012). This suggests a constructive feedback loop that supports robust T cell activation. Additionally, the transcription factor Twist1, alongside T-bet, contributes to Th1 cell development and functionality. These transcription factors can stimulate the production of miR-148a in Th1 cells, which is crucial for enhancing cell survival by decreasing the expression of the pro-apoptotic protein Bim. This mechanism is vital for maintaining the stability of Th1 cells in chronic inflammatory conditions (Haftmann et al., 2015). Finally, miR-301a has a promising role in the differentiation of Th17 cells by activating STAT3, achieved by inhibiting the expression of PIAS3, an inhibitor of STAT3 function (Levy et al., 2006). This finding underscores the potential of miR-301a in shaping Th17 cell responses and highlights its importance in immune regulation (Mycko et al., 2012).

Many miRNAs play a crucial and constructive role in understanding the immunopathogenesis of periodontal disease, particularly in relation to B cells. B cells account for a significant amount of the inflammatory infiltrate in diseased periodontal tissues, and larger levels of B lymphocyte infiltration are associated with advanced periodontitis (Stoufi et al., 1987). Interestingly, the expression of the five periodontal inflammation-related miRNAs linked with periodontal disease, miR-125, miR-148, miR-155, miR-181 and miR-217 (Stoecklin-Wasmer et al., 2012; Xie et al., 2011; Lee et al., 2011), is higher in advanced periodontal lesions. These five miRNAs affect a network of transcription factors, suggesting a link between periodontal disease development and B cell differentiation (Luan et al., 2018). miR-125b inhibits B cell terminal differentiation by downregulating transcription factors such as Blimp1 and IRF4.122, miR-148 suppresses BTB domain and CNC homologue 2 (Bach2) expression and promotes plasma cell lineage commitment (Pors et al., 2015). miR-148 also targets the proapoptotic proteins PTEN and Bim, promoting germinal center (GC) B cell survival (Gonzalez-Martin et al., 2016). miR-217 stabilizes the transcriptional repressor Bcl-6 in GC B cells, leading to increased class-switched antibodies and somatic hypermutation. This is achieved by down-regulating the expression of the DNA damage response and repair gene network (de Yebenes et al., 2014). Both miR-155 and miR-181 inhibit the production of activation-induced cytidine deaminase (AID), reinforcing and suppressing unregulated AID expression (Teng et al., 2008; de Yébenes et al., 2008). These studies highlight some of the miRNAs implicated in the innate and adaptive immune response to periodontal pathogens, as well as the early course of periodontal disease in neutrophils, macrophages, dendritic cells, and T and B lymphocytes.

## 4 Clinical and therapeutic applications of miRNAs in periodontal disease

Therapeutic medicines can be made from individual miRNAs. They have the ability to affect numerous regulatory networks and target multiple genes. Novel miRNA therapeutics have become more and more well-known as possible therapeutic agents in recent years due to their small size, ease of passage through tissues and membranes, microregulatory effects on gene expression, and capacity to simultaneously affect several related mRNAs (Diekwisch, 2016). Anti-miRs that target miR-122 to treat hepatitis and a mimic for the tumor suppressor miRNA miR-34 are examples of early clinical results for miRNA uses in disease treatment (Rupaimoole and Slack, 2017). Their potential utility in the treatment of periodontal disease is linked to the intricacy of the condition and the clinical applicability of miRNA mimics and anti-miRs to regulate the miRNA environment of host tissues and disease processes. When oral pathogens infect a host, their miRNA expression is altered through two main mechanisms: (i) pathogen-encoded miRNAs that mimic host miRNAs to help the pathogen reproduce and survive, and (ii) modifications in host miRNA expression levels as a component of the host immune response (Eulalio et al., 2012; Plaza, 2016). The host immunological response to infections and the advantages of bacterial infection are both impacted by the loss of the homeostatic balance of miRNA control. Therefore, there may be therapeutic promise for treating periodontal disease by modifying miRNA function to reduce excessive inflammation linked to tissue disintegration. miRNA mimics and miRNA antagonists can be used to either increase or decrease miRNA levels, respectively. Short double-stranded oligonucleotides that are chemically produced and functionally imitate a premiRNA complex are known as miRNA mimics (Wang, 2011; Goldgraben et al., 2015). On the other hand, miRNA antagonists are single-stranded oligonucleotides that are intended to functionally decrease miRNA activity by complementing miRNA sequences (Robertson et al., 2010; Stenvang et al., 2012). Not only do distinct bacteria control distinct host miRNAs, but they can also induce distinct miRNA reactions in various cell types in response to infection (Staedel and Darfeuille, 2013). Nonetheless, a group of essential miRNAs in response to bacterial infection has been identified using genome-wide miRNA transcriptional response research in human immune cells reacting to different pathogens (Siddle et al., 2015).

Studies of immunological disorders and tumor immune responses in animal models offer compelling proof of principle, despite the fact that therapeutic targeting of miRNAs for the treatment of periodontal disease is still in its infancy. Pathologic immunological diseases emerge as a result of immune responses triggered by subsets of activated T cells. For instance, Th2 cells mediate humoral immunity and elicit allergy immunological responses, whereas Th1 cells enhance cellular immunity and influence the onset of autoimmune disorders (Skapenko et al., 2005). By suppressing the effector activity of Th2 cells and preventing house dust mite-induced allergic airway disorders in animal lungs, intranasal administration of miR-126 or miR-145 antagonists addressed the Th2 cell-mediated allergic immune response (Collison et al., 2011; Mattes et al., 2009). It has been shown in previous research that miR-326 controls Th17 cell differentiation. In mice, lentiviral-based *in vivo* suppression of miR-326 reduced EAE and prevented Th17 development (Du et al., 2009; Luck et al., 2015). Furthermore, retroviral vector-induced overexpression of miR-10 restricted differentiation into the Th17 subset of Th cells and prevented inducible Treg cells from becoming follicular Th cells. Therefore, supplementing with miR-10 had a protective effect and delayed the onset of autoimmune encephalomyelitis (Takahashi et al., 2012).

Using an *in vitro* and rat model, a recent study investigated whether miR-200c can reduce inflammation and alveolar bone resorption in periodontitis. In HGFs, the overexpression of miR-200c dramatically decreased IL-6 and eight and suppressed interferon-related developmental regulator-1 (IFRD1). Additionally, miR-200c downregulated p50 and p65 (Akkouch et al., 2019). In a rat model of periodontitis, local injection of miR-200c markedly increased the expression of miR-200c in the gingiva and decreased IFRD1, IL-6, IL-8, and the ratio of osteoprotegerin to receptor activator of NF-κB. These results suggest that miR-200c may be a novel strategy for preventing periodontitis and related bone loss.

Exosomes are small secretory organelles that can be released from cells into the extracellular environment. They carry a variety of signal molecules, including proteins and miRNAs (Pegtel and Gould, 2019). Numerous exosomal miRNAs have been shown to play distinct roles in periodontitis (Atsawasuwan et al., 2018; Shen et al., 2020; Nik Mohamed Kamal NNS et al., 2020; Lv et al., 2020; Han et al., 2020). For instance, tiny extracellular vesicles from patients with periodontitis exhibit overexpression of miR-140-5p, −146a-5p, and -628-5p, which may be biomarkers (Han et al., 2020). Dental pulp stem cells’ exosomal miR-1246 encourages periodontal tissues to change from a proinflammatory to an anti-inflammatory phenotype (Shen et al., 2020). In mice with periodontitis caused by LPS, exosomal miR-200c reduces proinflammatory cytokines (Krongbaramee et al., 2021). A recent study showed that in experimental periodontitis-affected mice, PDLSC-derived exosomal miR-31-5p targets eNOS to control alveolar bone repair (Lu et al., 2023). The osteoclast genes NFATc1, TRAF6, and c-Fos were increased by exosomes that overexpressed miR-31-5p, while they were downregulated by exosomes that repressed miR-31-5p. However, the lack of a comprehensive investigation into the relationship between miR-31-5p and macrophage osteoclast differentiation *in vitro*, limits this study (Lu et al., 2023). Future research should examine the relationship between miR-31-5p and eNOS in more detail and use *in vivo* tests to examine the role of miR-31-5p in alveolar bone homeostasis.

Another study clarifies the impact of Treg cell-derived exosomes (Treg-Exos), especially those containing miR-21, on PDLSC osteogenic development and periodontal tissue regeneration (Xia et al., 2025). Treg cells and Treg-Exos were found to significantly enhance the osteogenic differentiation of PDLSCs *in vitro*, as demonstrated by elevated Runx-2/Osterix expression, improved mineralization, and increased ALP activity. PDLSC osteogenesis was further stimulated by exosomes produced from Treg cells overexpressing miR-21, while exosomes with miR-21 knockdown showed an inhibitory impact. Treg-Exos infusion reduced periodontal disease and enhanced tissue structure *in vivo*.

In a study, the osteogenic role of periodontal ligament fibroblasts-derived exosomes generated by PGE2 was identified on PDLSCs (Lin et al., 2022). MiR-34c-5p was shown to be increased in exosomes-PGE2 compared to exosomes-normal using high-throughput miRNA sequencing. Overexpression of miR-34c-5p decreased ERK1/2 phosphorylation and hindered osteogenic differentiation. Furthermore, miR-34c-5p was found to target special AT-rich sequence-binding protein 2 (SATB2) using a dual-luciferase reporter experiment. Exosomal miR-34c-5p was found to suppress PDLSC osteogenic development through the SATB2/ERK pathway. Another study was conducted to examine the amounts of differentially expressed exosomal RNAs before and after treatment in both healthy individuals and patients with periodontitis (Kwon et al., 2023). It was shown that two differentially expressed exosomal miRNAs (miR-1304-3p and miR-200c-3p) were frequently downregulated during periodontitis and returned to normal levels following treatment. The top three target genes that are frequently controlled by differentially expressed miRNAs were found to be *NR3C1, GPR158,* and *CNN3*.

A transcription factor called X-box binding protein 1 (XBP1) is produced by unfolded proteins and alternative splicing of the endoplasmic reticulum.30 In inflammatory disorders, many miRNAs can control XBP1. It has been established that periodontitis-affected tissues have higher levels of XBP1 expression (Wang et al., 2016; Lundmark et al., 2018). In LPS-induced PDLSCs and associated exosomes, miR-205-5p was found to be downregulated (Kang et al., 2023). Exo-miR-205-5p reduced the percentage of Th17 cells in LPS-treated rats, prevented inflammatory cell infiltration, and reduced the production of TNF-α, IL-1β, and IL-6 (Kang et al., 2023). Furthermore, miR-205-5p targeted XBP1. The effects of exo-miR-205-5p on reducing inflammation and controlling Treg/Th17 balance in LPS-induced cells were lessened by overexpression of XBP1 (Kang et al., 2023).

Anti-tumor immunotherapies have also been studied in relation to miR-based immune responses. *In vivo* miR-138 augmentation inhibited glioma cell proliferation and extended the longevity of glioma-affected rats (Wei et al., 2016). To increase the miR-155 activity in an ovarian cancer microenvironment, nanoparticles containing miR-155 mimics as cargo were introduced into DCs *in vivo* in another study (Cubillos-Ruiz et al., 2012). This caused DCs to change from immunosuppressive to immunostimulatory cells and activate strong anti-tumor immune responses (Zhang et al., 2013).

In recent years, over a dozen miRNA delivery systems-mostly viral and non-viral-have been developed. Retroviruses, lentiviruses, and adenoviruses are the key players in viral miRNA delivery systems; nevertheless, these systems might cause a robust immune response, which may lessen their efficacy (Zhang et al., 2013; Yang et al., 2015; Mishra et al., 2016). However, non-viral methods have the challenging task of moving miRNAs or some of their antagonists across the cell membrane while maintaining their structural integrity on the way to the nucleus (Mishra et al., 2016). Lipid-based systems (like liposomes) and polymer-based strategies (like polyethylenimine (PEI), poly (lactic-co-glycolic acid) (PLGA), and poly (amidoamine) (PAMAM)) are examples of these non-viral miRNA delivery techniques. Other recently created miRNA carriers include collagen, chitosan, protamine, and nanoparticles based on gold, iron, and silica (Mishra et al., 2016; Xavier et al., 2015; Gaharwar et al., 2014; Munagala et al., 2016). The majority of miR-based treatments administered *in vivo* use systemic injection, which is costly, ineffective, and prone to negative side effects. Thus, efforts are underway to develop targeted miRNA delivery platforms that enhance the homing of delivery vehicles to particular tissues.

One obstacle to miR-based therapy is the intrinsic instability of miRNA in blood. Unmodified miRNA with an unchanged 2′OH is rapidly broken down by nucleases, especially ribonucleases (RNase). Furthermore, the half-life of naked miRNA in circulation is only a few minutes to an hour because of rapid renal excretion clearance (Wang et al., 2025). Achieving accurate targeted administration with efficient penetration which depends on the capacity to cross cell membranes—is the main challenge in miRNA treatment. This problem is caused by miRNAs’ hydrophilic properties and negative charge, which lead to charge repulsion and restricted permeability (Wang et al., 2025). The main mechanism of miRNA uptake is the endocytic pathway. A range of nucleases in the lysosome, where the pH is between 5 and 6, break down miRNA when it becomes stuck inside endosomes (Wang Y. et al., 2020). Therefore, encouraging endosomal escape and guaranteeing the cytosolic distribution of medicines are essential for a successful miR-based therapy. Under normal circumstances, miRNA binds to the 3′UTR of target mRNA to control gene expression. Nevertheless, it has been discovered that miRNA can also attach to the coding sequence (CDS) or 5′UTR, producing off-target effects (Diener et al., 2022). This may lead to a number of undesirable biological consequences, such as the control of cell division, apoptosis, proliferation, and other processes (Wang et al., 2025). Single-stranded RNAs can be identified by the host system as pathogens, triggering the innate immune system (Munagala et al., 2016). Significant inflammatory cytokine production and TLR activation of the IFN pathway may follow miRNA injection into the body (Sharma et al., 2022). The toxicity of delivery carriers with very positive charges is another consideration. Nanoparticles can cause immunological reactions because they are perceived as foreign substances when they enter the body.

These preclinical and clinical investigations collectively demonstrate the huge therapeutic promise of miR-based approaches. The use of miR-based therapies in conjunction with existing periodontal treatment strategies may be one of the therapeutic methods for the treatment of periodontal disease. Future studies will determine the best ways to treat and reverse periodontal disease by combining certain miRNA combinations and dosages with appropriate carrier vehicles and delivery methods.

## 5 Diagnostic applications of miRNAs in periodontal disease

Currently, the most prevalent molecular biomarkers for periodontitis found in oral fluid can be divided into three main groups: tissue breakdown products, inflammatory mediators and host response modifiers, and host-derived enzymes and their inhibitors (Li and Kowdley, 2012; Cheng et al., 2014; Chen et al., 2008; Mitchell et al., 2008). Although they are unable to forecast the onset and early progression of periodontal disease, these molecular biomarkers can identify the existence and severity of the condition. Recently, a new class of extremely sensitive and specific biomarkers known as miRNAs has surfaced (Robbins and Morelli, 2014). Because of their packaging (Fernández-Messina et al., 2015; de Candia et al., 2016; Correia et al., 2017) that keeps them stable in extracellular fluids, miRNAs are perfectly suited to function as non-invasive indicators for periodontal disease.

Oral bacteria and inflammatory diseases impair the immunological and epithelial cells’ ability to function and dysregulate the expression of miRNA in these cells in periodontal disease. Both immune and non-immune cells actively release miRNAs into extracellular fluids and other surroundings in addition to synthesizing them intracellularly (Schmalz et al., 2016; Yoneda et al., 2019; O’Brien J. et al., 2018). The released miRNAs are encased in lipid vesicles or linked to high-density lipoproteins or RNA-binding proteins. In extracellular fluids, they exhibit a high degree of stability (Gupta, 2012). Five miRNAs including miR-142-3p, miR-146a, miR-155, miR-203, and miR-223, have been suggested as indicators of periodontal disease, regardless of their biological roles (Gupta, 2013). Also, miR-21-3p stands out due to its connections with adhesion molecules, T-lymphocyte receptors, and the MAPK tumor signaling pathway, indicating its multifaceted role in cellular processes (Saito et al., 2017). Furthermore, miR-146 and miR-155 serve as crucial regulators of the immune system, collaboratively enhancing the expression of specific cytokines such as RANKL, type I and type II interferons (IFN), IL-1, and TNF-α (Jeker and Bluestone, 2013). This suggests a significant link between these miRNAs and chronic inflammation. Lastly, miR-200 plays an important role in the mesenchymal–epithelial transition by regulating the expression of the transcription factor ZEB-1 (Wong, 2012).

Due to their close proximity to the periodontium and their potential for non-invasive sample collection, gingival crevicular fluid (GCF) and saliva have been regarded as the best sampling conditions for periodontal diagnostics (Zhang et al., 2000; Byun et al., 2015). More than 600 miRNAs have been found through miRNA profiling in the GCF of healthy individuals or patients with periodontitis. The most significantly expressed miRNA among these was miR-223, which was similarly increased in periodontitis samples (Diekwisch, 2016). In addition to GCF, saliva also contains salivary gland secretions, oral mucosal exudates, and a possible pool of biological markers in a hypotonic environment, making it more complex than GCF alone. Salivaomics has been proposed as a concept that includes miRNA profiles, proteomics, metabolomics, transcriptomics, and genomes. For the diagnosis of oral cancer, pre-cancer, and oral lichen planus disease, miRNA profiling in saliva has been studied (Rupaimoole and Slack, 2017; Eulalio et al., 2012; Plaza, 2016).

In a most recent study, miRNAs-21, -29b, −34a, −126, −132, −146a, and −221 were detected in the GCF of 24 teenage patients (less than 18 years old) receiving treatment with a full-mouth multibracket appliance (Rolfes et al., 2025). Before, 7 days, 5 weeks, and 3 months following the application of orthodontic force, non-invasive GCF samples were obtained from the second premolar in each jaw. Prior to treatment, samples from the mandible and maxilla showed a moderate to high correlation and all examined miRNAs were consistently detectable in the GCF. With orthodontic tooth movement, all miRNAs displayed altered expression levels.

Mice infected with *Fusobacterium nucleatum* displayed a marked increase in alveolar bone resorption and 100% bacterial colonization on the gingival surface. Thirteen miRNAs were downregulated and seven were increased in the 16-week infection group. Notably, there was differential expression of miR-205, miR-210, and miR-199a-3p at 8 weeks and miR-28 at 16 weeks, all of which have been linked to human periodontitis (Jeepipalli et al., 2025b). Furthermore, miR-126-5p has been found to be a possible biomarker for periodontal disease patients (Jeepipalli et al., 2025b). The mandibles of mice infected with *S. gordonii* were examined for differentially expressed miRNAs (Aravindraja et al., 2024). Alveolar bone resorption and gingival colonization by *Streptococcus gordonii* were verified. At 8 weeks, miRNA profiling of the mandibles of *S. gordonii*-infected mice showed 22 downregulated miRNAs (miR-133, miR-1224) and 191 upregulated miRNAs (miR-375, miR-34b-5p). On the other hand, 32 miRNAs (miR-1937c, miR-720) were downregulated and 10 miRNAs (miR-1902, miR-203) were increased at 16 weeks after infection. In the mandibles of infected mice, two miRNAs, miR-210 and miR-423-5p, were frequently elevated, while miR-2135 and miR-145 were frequently downregulated (Aravindraja et al., 2024). Another study revealed that the infection of *T. forsythia* downregulate two miRNAs, miR-375 and miR-200c (Aravindraja et al., 2023a). The gingival tissue and saliva of patients with periodontitis have also been shown to have six downregulated miRNAs in the 8-week infection (miR-200b, miR-141, miR-205, miR-423-3p, miR-141-3p, miR-34a-5p) and two downregulated miRNAs in the 16-week infection (miR-27a-3p, miR-15a-5p) (Aravindraja et al., 2023a). Also, three distinct miRNAs—miR-486, miR-126-3p, and miR-126-5p, together may function as an invasive biomarker of *Treponema denticola*-infection in periodontal disease (Aravindraja et al., 2023b). During an 8-week *P. gingivalis* infection, the miRNA profiling revealed 26 upregulated miRNAs (such as miR-804, miR-690) and 14 downregulated miRNAs (such as miR-1902, miR-1937a); during a 16-week infection, seven upregulated miRNAs (such as miR-145, miR-195) and one downregulated miR-302b) (Aravindraja et al., 2023c). Another study used high-throughput Nanostring nCounter miRNA expression panels to assess differential mandibular miRNA profiles using partial human mouth microbes (PAHMM) (*S. gordonii, F. nucleatum, P. gingivalis, T. denticola*, and *T. forsythia*) in a polybacterial periodontal infection mouse model (Aravindraja et al., 2022). There are seven miRNAs in both sexes that may be involved in the pathophysiology of periodontitis: miR-9, miR-148a, miR-669a, miR-199a-3p, miR-1274a, miR-377, and miR-690.

A recent genome-wide miRNA expression study in 16 gingival tissue samples found 177 deregulated miRNAs. The results showed high expression of miR-140-3p and -145-5p, while the levels of miR-125a-3p were observed to be significantly lower in inflamed tissues (Buragaite-Staponkiene et al., 2023). After a comprehensive validation, four miRNAs, namely, miR-140-3p, -145-5p, −146a-5p, and -195-5p, were selected for further study in a larger sample of salivary and blood plasma specimens. Bleeding was linked to elevated salivary miR-145-5p levels. Participants with periodontal disease had greater plasma-derived levels of miR-140-3p.

Exosomes have the potential to diagnose and treat human diseases because they are essential for chemical exchange and signal communication between cells (Kang et al., 2023). Considering potential indicators, miR-103a-3p, -126-3p, and -150-5p are downregulated in plasma exosomes in periodontitis.9 Exosomes produced from LPS-induced PDLSCs have reduced expression of miR-155-5p, which contributes to the progression of chronic periodontitis (Zheng et al., 2019). Exosomes produced from LPS-treated PDLSCs also showed downregulation of miR-205-5p, which is consistent with that observed in LPS-treated PDLSCs (Kang et al., 2023).

However, these findings raise the question if these miRNAs could serve as diagnostic markers for periodontal diseases or simply reflect an elevated inflammatory state associated with different disorders. Another concern is the vast diversity of results. Although many miRNAs were detected, convergence between the experiments, particularly *in vivo*, is minimal, resulting in inconsistent outcomes. Investigations of periodontal disease may differ for a variety of reasons. On the one hand, modest variability in patient selection and diverse clinical procedures likely explains these disparities. On the other hand, changes in methodologies utilized for miRNA detection could play a critical effect. According to a study of the techniques employed, miRNA detection processes were quite comparable. Nonetheless, it is clear that there are several variations in certain processes. Another element that could be essential for the varying results is the small number of study participants. The use of small sample sizes makes research more susceptible to a variety of biases and errors, which could lead to inconsistent study outcomes and possibly false-positive results. There is undoubtedly a complicated explanation for the variations in the outcomes. Different methods, clinical criteria, small groups of patients, and small specificity of miRNAs for periodontal disease could be causal complex.

Also, different internal controls, methodologies for miRNA isolation, statistical analysis, and types of saliva (stimulated/unstimulated, whole saliva/supernatant) were utilized. Interestingly, compared to studies on periodontitis, the number of study participants in these investigations is already bigger, but they are also varied, with some small group sizes. Additionally, the kind of saliva, particularly whether stimulated or unstimulated, as well as the precise method of saliva collection and patient selection criteria may be significant. Furthermore, unstable exogenous miRNAs in saliva may cause rapid changes in miRNA concentrations from bacteria and inflammatory reactions (Park et al., 2009). The detectability of miRNAs in human saliva samples may be potentially impacted by their possible concentrations in bodily fluids, such as exosomes (Bahn et al., 2015). In addition, it was noted that saliva contains vesicle-free noncoding RNAs (Gallo et al., 2012). Furthermore, it is unclear if miRNAs found in tissue studies could serve as markers for salivary diagnostic techniques. The observation of radically distinct miRNA expression across malignant tissue and bodily fluids (Momen-Heravi et al., 2014) could be comparable for periodontitis. The dependability of study results is also thought to be limited by the relative size of the sample and external factors like as age, gender, ethnicity, and habit effect. The variables include financial and infrastructure facility concerns to undertake large scale investigations for getting a definitive conclusion and the difficulty in pinpointing the precise pinpoint origin of miRNA need more justification (Rapado-González et al., 2018). Further investigations of miRNAs in GCF or saliva and their association with periodontal diseases may give innovative diagnostic tools for the early detection of periodontal disease and treatment plan monitoring.

## 6 Conclusion

The immune system’s response to bacteria that accumulate along the gum line can lead to periodontal disease, an inflammatory condition that may progress to tooth loss and looseness in more severe stages. miRNAs are crucial players in the development of periodontal disease, as they interact with the immune system and various periodontal cells, including GECs and periodontal ligament cells. By influencing several aspects of the immune response, these miRNAs present promising opportunities for targeted therapies. Exploring these miRNAs as potential therapeutic targets could pave the way for more effective treatments for periodontal disease, ultimately improving oral health outcomes. This review paper emphasizes the important role of miRNAs in the immunopathogenesis of periodontal disease. It proposes that utilizing RNA mimics and antagomirs could offer innovative therapeutic strategies for more effective treatment of periodontal disease. This approach holds great potential for enhancing patient outcomes and advancing our understanding of periodontal health. To enhance our understanding of the pathobiology of periodontal diseases, it is vital to build on our preliminary findings that demonstrate differential expression of several miRNAs between “healthy” and “diseased” gingival tissues. By refining the expression patterns in relation to specific cell populations and conducting detailed mechanistic studies, we can deepen our insights into their roles. Utilizing integrated bioinformatics techniques to analyze miRNA expression and reconstruct regulatory networks will significantly advance our knowledge in this area. This collaborative effort can pave the way for innovative approaches to better diagnose and treat periodontal diseases. Future research findings will be helpful in creating other therapeutic strategies, particularly those that treat periodontal disease by using miRNA delivery methods.
